# Valproic Acid Sensitizes Hepatocellular Carcinoma Cells to Proton Therapy by Suppressing NRF2 Activation

**DOI:** 10.1038/s41598-017-15165-3

**Published:** 2017-11-08

**Authors:** Jeong Il Yu, Changhoon Choi, Sung-Won Shin, Arang Son, Ga-Haeng Lee, Shin-Yeong Kim, Hee Chul Park

**Affiliations:** 1 Departments of Radiation Oncology, Samsung Medical Center, Seoul, 06351 South Korea; 2 Sungkyunkwan University School of Medicine, Seoul, 06351 South Korea; 3 Department of Medical Device Management and Research, SAIHST, Seoul, 06351 South Korea

## Abstract

Although efficacy of combined histone deacetylase (HDAC) inhibitors and conventional photon radiotherapy is being tested in clinical trials, their combined effect with proton beam radiotherapy has yet to be determined. Here, we compared combined effect of valproic acid (VPA), a class I and II HDAC inhibitor and antiepileptic drug with proton and photon irradiation in hepatocellular carcinoma (HCC) cells *in vitro* and *in vivo*. We found that VPA sensitized more Hep3B cells to proton than to photon irradiation. VPA prolonged proton-induced DNA damage and augmented proton-induced apoptosis. In addition, VPA further increased proton-induced production of intracellular reactive oxygen species and suppressed expression of nuclear factor erythroid-2-related factor 2 (NRF2), a key transcription factor regulating antioxidant response. Downregulation of NRF2 by siRNA transfection increased proton-induced apoptotic cell death, supporting NRF2 as a target of VPA in radiosensitization. In Hep3B tumor xenograft models, VPA significantly enhanced proton-induced tumor growth delay with increased apoptosis and decreased NRF2 expression *in vivo*. Collectively, our study highlights a proton radiosensitizing effect of VPA in HCC cells. As NRF2 is an emerging prognostic marker contributing to radioresistance in HCC, targeting NRF2 pathway may impact clinical outcome of proton beam radiotherapy.

## Introduction

The reversible acetylation of histones is one of the key modifications for the epigenetic control of gene expression^1,2^ and is regulated by the reciprocal action of histone acetyl-transferases (HATs) and histone deacetylases (HDACs)^3^. There are 18 human HDACs, which can be divided into four classes based on the homology with yeast proteins. Class I, II and IV HDACs are classical HDACs with Zn^2+^-dependent active site whereas class III HDACs are a family of NAD^+^-dependent enzymes. Altered expression of HDACs and unbalanced acetylation of histones and non-histone proteins, however, are frequently detected in certain human cancers^4–6^. Thus, HDACs have been considered as therapeutic target for cancer treatment^7–9^.

Various HDAC inhibitors with different specificity to HDACs have been tested for antineoplastic activity^10^. Four HDAC inhibitors, vorinostat, romidepsin, panobinostat and belinostat are now approved for the treatment of cutaneous T-cell lymphoma and other HDAC inhibitors are being clinically tested for other types of cancer. Nonetheless, the clinical outcomes of HDAC inhibitors as a single agent to treat solid tumors are not satisfactory. Therefore, the combination strategy of HDAC inhibitors with other anticancer treatments including targeted therapy and radiation therapy (RT) is another worthy of consideration^4^.

RT is one of the main modalities for cancer treatment and kills cancer cells using ionizing radiation such as photon and particle beams^11–13^. Growing evidence shows that HDAC inhibitors sensitize human cancer cells to ionizing radiation^14–17^. One of the mechanisms of radiosensitization by HDAC inhibitors is to increase RT-mediated cell death by modulating DNA damage repair signaling; ionizing radiation generates DNA double-strand breaks (DSB) and HDACs downregulate DSB repair genes such as Ku70, Ku80, RAD51 and DNA-dependent protein kinase^14–17^. On the basis of the pre-clinical evidence, several clinical trials have been in progress. Pelvic radiation and vorinostat (PRAVO) study was the first clinical trial of combination of HDAC inhibitors with palliative RT confirming the safety and tolerability of the combination^18^. A retrospective study of valproic acid (VPA) treatment during RT for glioblastoma showed association of VPA and improved survival^19^. A phase 2 study of combination of RT, temozolomide and VPA for glioblastoma revealed combined VPA/RT/temozolomide was well tolerated and expected to improve outcomes^20^.

Particle beams such as protons and carbon ions have different biological activities compared with the same physical dose of photon, so called relative biological effectiveness (RBE). Proton beam shows superior dose distribution compared with photon due to “Bragg peak” and has similar or slightly higher RBE as 1.1 than the photon^21^. However, the RBE of proton could be changed by several circumstances, like energy of proton, type of irradiated tissue, and dose/fraction. It is also reasonable to expect that RBE could be modified by the addition of chemotherapeutic agents^22^.

VPA is a well-known anti-epileptic drug that has been used for about 50 years to treat seizure and inhibits class I and IIa HDACs and its usage in cancer treatment has been recently described^5,23^. Synergistic or additive effect of VPA with conventional photon therapy has been extensively studied in various cancers^24–28^, but combination effect of VPA with proton therapy has yet to be determined. Using clinical radiation therapy machines, we report here for the first time that VPA acted as proton radiosensitizer in hepatocellular carcinoma (HCC) cells *in vitro* and *in vivo*. As proton beam therapy is a favorable option for HCC treatment, our findings suggest that VPA may have benefit in treating HCC with proton therapy.

## Results

### VPA treatment inhibits deacetylation of histones and minimally reduces cell proliferation

**Figure 1:**
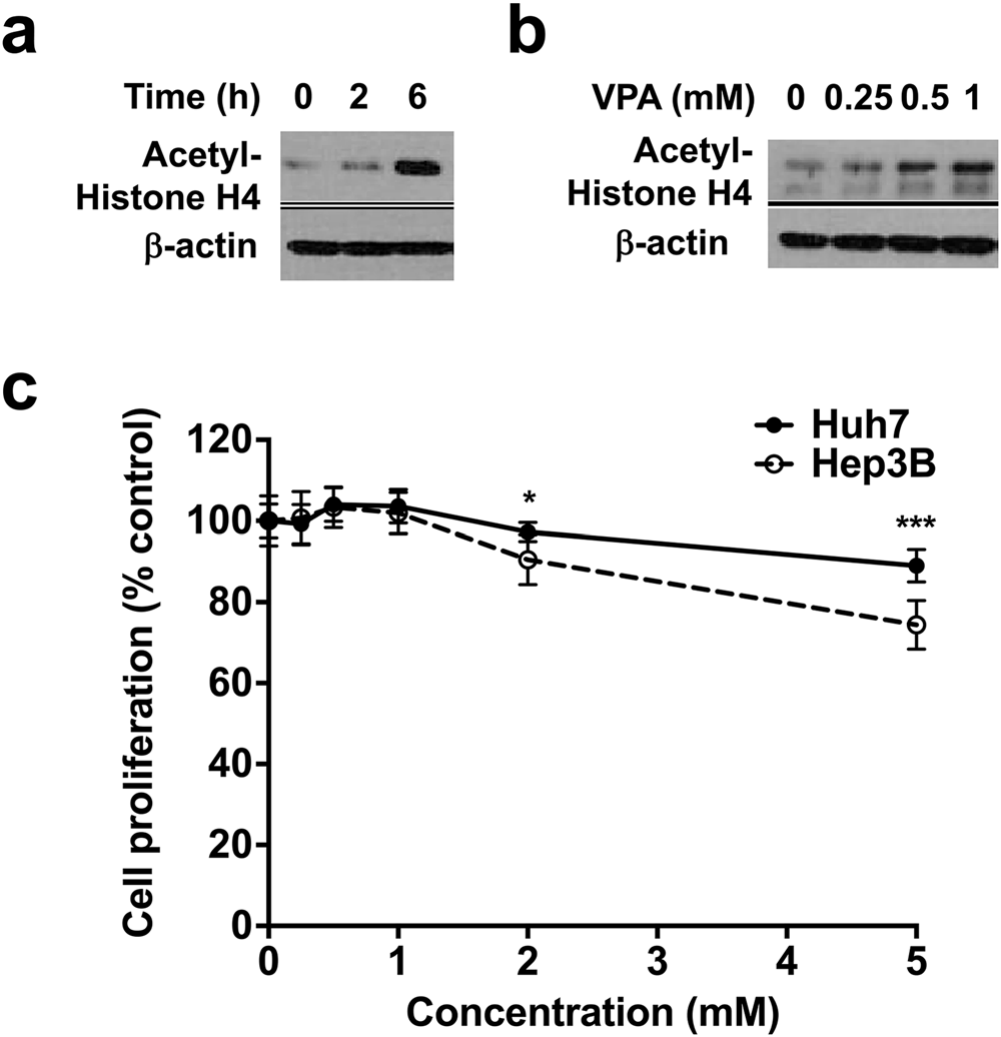
Valproic acid (VPA) inhibits histone deacetylase activity in human hepatocellular carcinoma cells. (**a**) Western blot analysis shows time-dependent increase of histone H4 acetylation after 1 mM VPA treatment in Hep3B cells. (**b**) VPA increased histone H4 acetylation in concentration-dependent manner. Samples were harvested 72 h after treatment of indicated concentrations of VPA in Hep3B cells. (**c**) VPA treatment leads to a modest decrease in proliferation of human hepatocarcinoma Huh7 and Hep3B cells. After 48 h of treatment of indicated concentrations of VPA, cell proliferation was determined using MTT assay. Data are mean values ± SD of 8 samples. *p < 0.05, ***p < 0.001. The cropped blots are presented and their full-length blots are included in the Supplementary Fig. S1.

To test whether VPA, an antiepileptic drug, acts as HDAC inhibitor in human HCC cells, we determined acetylation of histone H4 after VPA treatment in Hep3B. Western blot analysis showed that acetylated histone H4 was low in untreated cells and increased at 6 h after 1 mM VPA treatment (Fig. 1a). VPA treatment also increased the acetylation of histone H4 in a concentration-dependent manner (Fig. 1b), confirming function of VPA as HDAC inhibitor in HCC cells. Next, the effect of VPA on HCC cell proliferation was assessed by using the colorimetric MTT metabolic activity assay. As VPA concentration increased, cell proliferation slightly decreased; 5 mM VPA reduced Huh7 and Hep3B cell proliferation by 11.0% and 25.6%, respectively, which suggests that Hep3B cells are more sensitive to VPA than Huh7 cells (*p* < 0.001) (Fig. 1c).

### VPA increases sensitivity of HCC cells to proton irradiation

**Figure 2:**
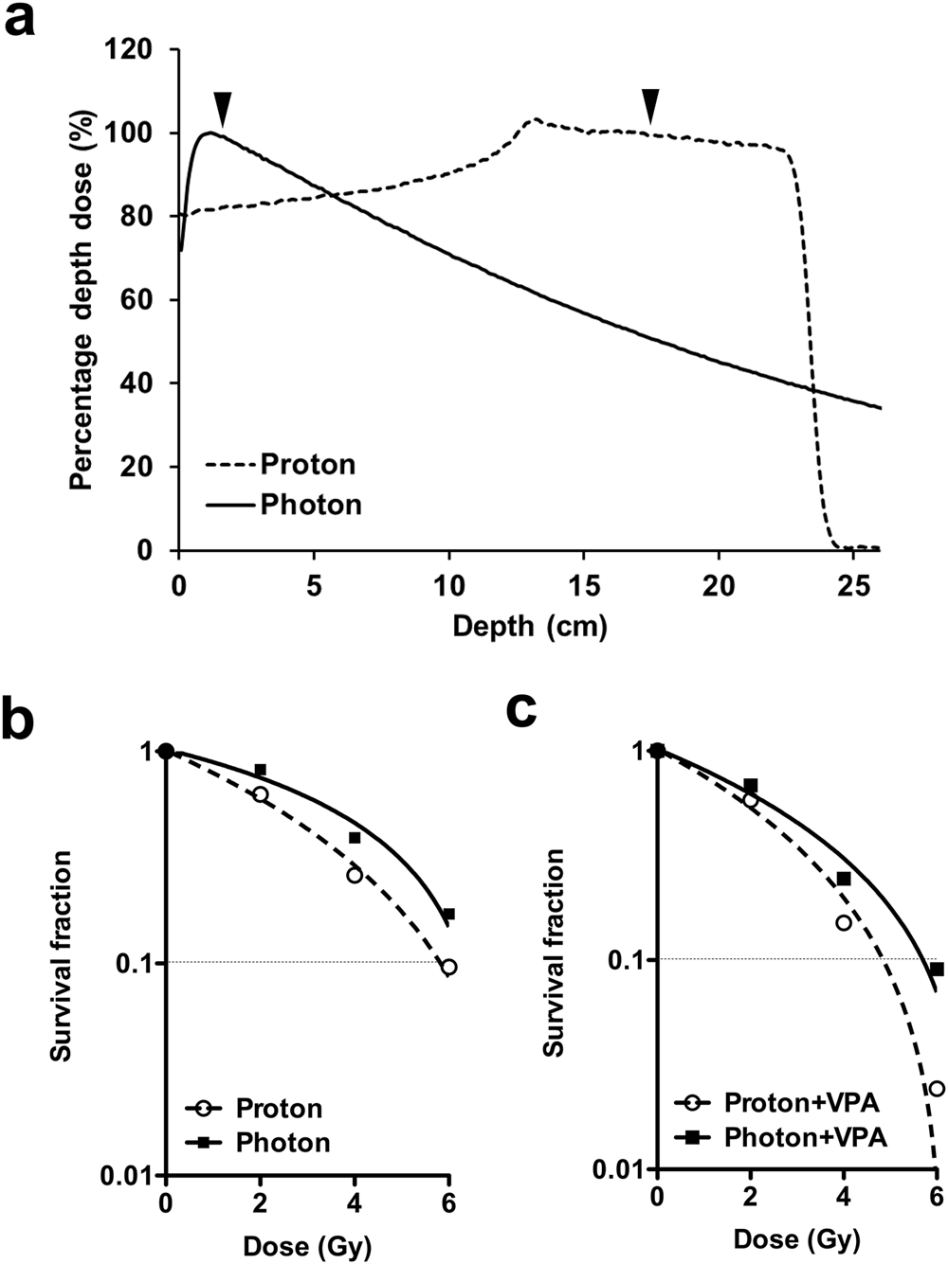
VPA sensitizes Hep3B cells to proton and photon irradiations. (**a**) Percentage depth dose graphs in water for photon (solid line) and proton beams (dashed line) indicate different energy distributions. Each arrowhead points to the positions at which cell plates were placed for photon or proton irradiation. (**b** and **c**) Clonogenic assay was performed to compare radiation sensitivity. Cells were seeded and irradiated with indicated doses of photon or proton beam with or without 1 mM VPA. After 15 days, survived colonies (>50 cells) were stained and counted. Representative dose-response curves are presented. Data are mean values ± SD of three samples. (**b**) Proton vs photon; (**c**) Proton + VPA vs photon + VPA.

To compare the potential of VPA in sensitizing HCC cells to photon and proton irradiation, we performed clonogenic survival assay. Considering that photon and proton beams have different dose distribution profiles, HCC cells were positioned at water-equivalent depth of 2 cm (for photon irradiation) or 17.2 cm (for proton irradiation, in the middle of the spread-out Bragg peak) using water-equivalent solid phantoms (Fig. 2a). The Hep3B cells were pre-treated with 1 mM VPA for 3 h and then were irradiated with the same physical doses of photon or proton beam. The cell survival curves were fit to a linear-quadratic model. Comparison of survival curves of proton and photon-irradiated cells showed that proton killed more Hep3B cells than photon (Fig. 2b). Proton RBE of Hep3B was 1.08 at survival fraction (SF) of 0.1, which is close to generic RBE of 1.1 that is being used in the clinic. VPA increased radiosensitivity with both proton and photon irradiation, but it killed more Hep3B cells when combined with proton. Value of SF at 6 Gy (SF6) of photon and VPA was much less than that of SF6 of proton and VPA (0.090 vs 0.024). VPA treatment increased proton RBE from 1.08 to 1.17 (Fig. 2c), suggesting a potential role of VPA in proton sensitization.

### VPA enhances proton irradiation-induced cell cycle arrest

**Figure 3:**
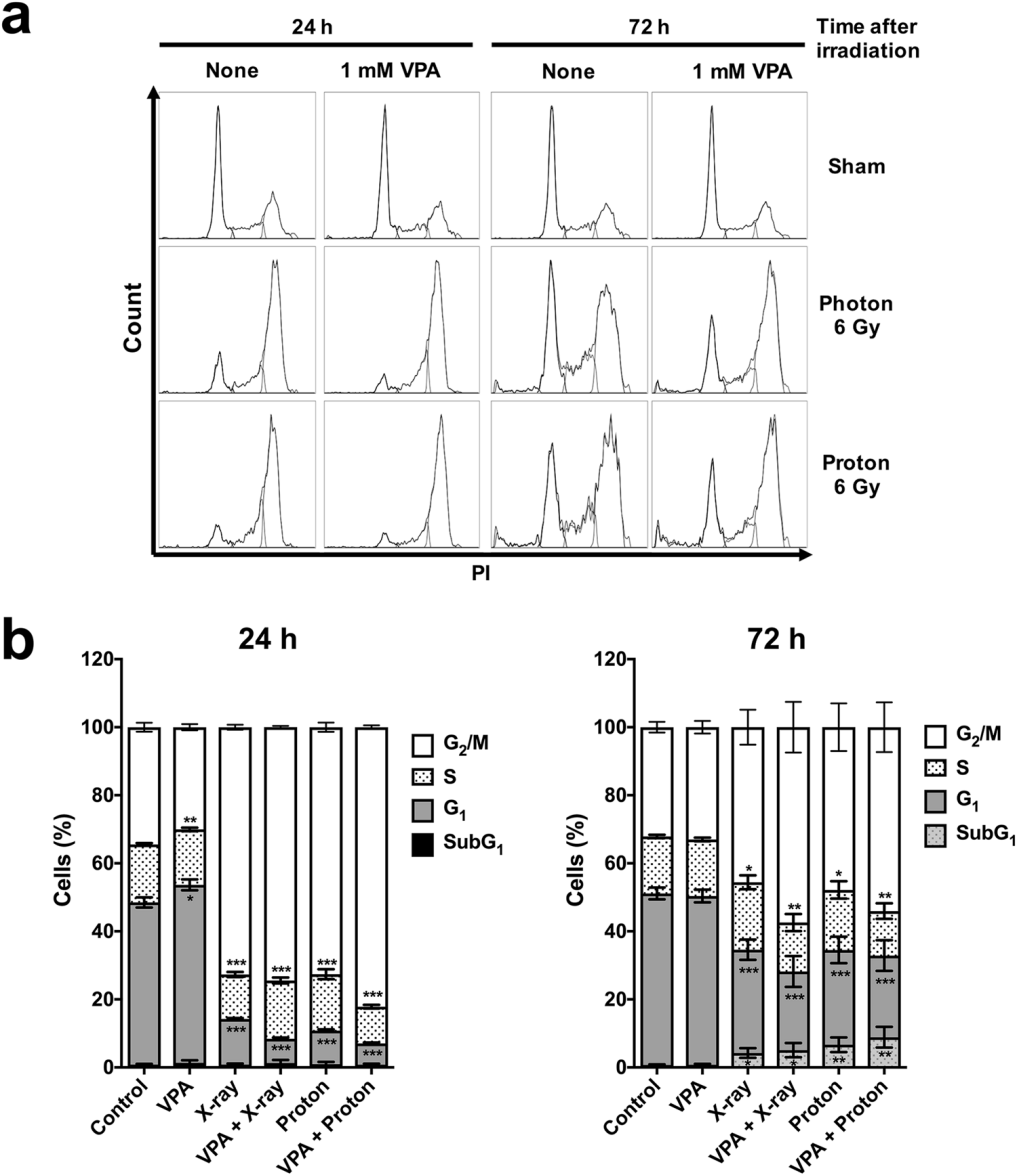
VPA attenuates radiation-induced G2/M arrest in Hep3B cells. (**a**) DNA histogram plots of Hep3B treated with or without 1 mM VPA at 24 h and 72 h after 6 Gy proton or photon irradiation. Cell cycle was assessed by propidium iodide staining and flow cytometry. (**b**) Distribution of each cell cycle shown as stacked column indicated attenuation of radiation-induced G2/M arrest by VPA. *p < 0.05, **p < 0.01, ***p < 0.001.

To understand how VPA potentiates proton radiosensitization in HCC cells, we first evaluated effect of VPA on cell cycle progression in Hep3B cells (Fig. 3). The majority of untreated cells stayed in G1 phase, which was minimally affected by 1 mM VPA alone. At 24 h post-irradiation, percentage of cells in the G2/M phase increased from 32.9% to 71.4% for photon and 70.1% for proton (*p* < 0.001) and then decreased to 45.8% and 49.2% at 72 h post-irradiation (*p* < 0.05). When combined with 1 mM VPA, percentage of G2/M arrested cells increased to 73.4% and 80.1% at 24 h (*p* < 0.001) and 59.9% and 58.6% at 72 h (*p* < 0.001), which was higher than radiation alone. It suggested that VPA may attenuate recovery from radiation-induced cell cycle arrest.

### VPA attenuates repair of proton-induced DNA damages

**Figure 4:**
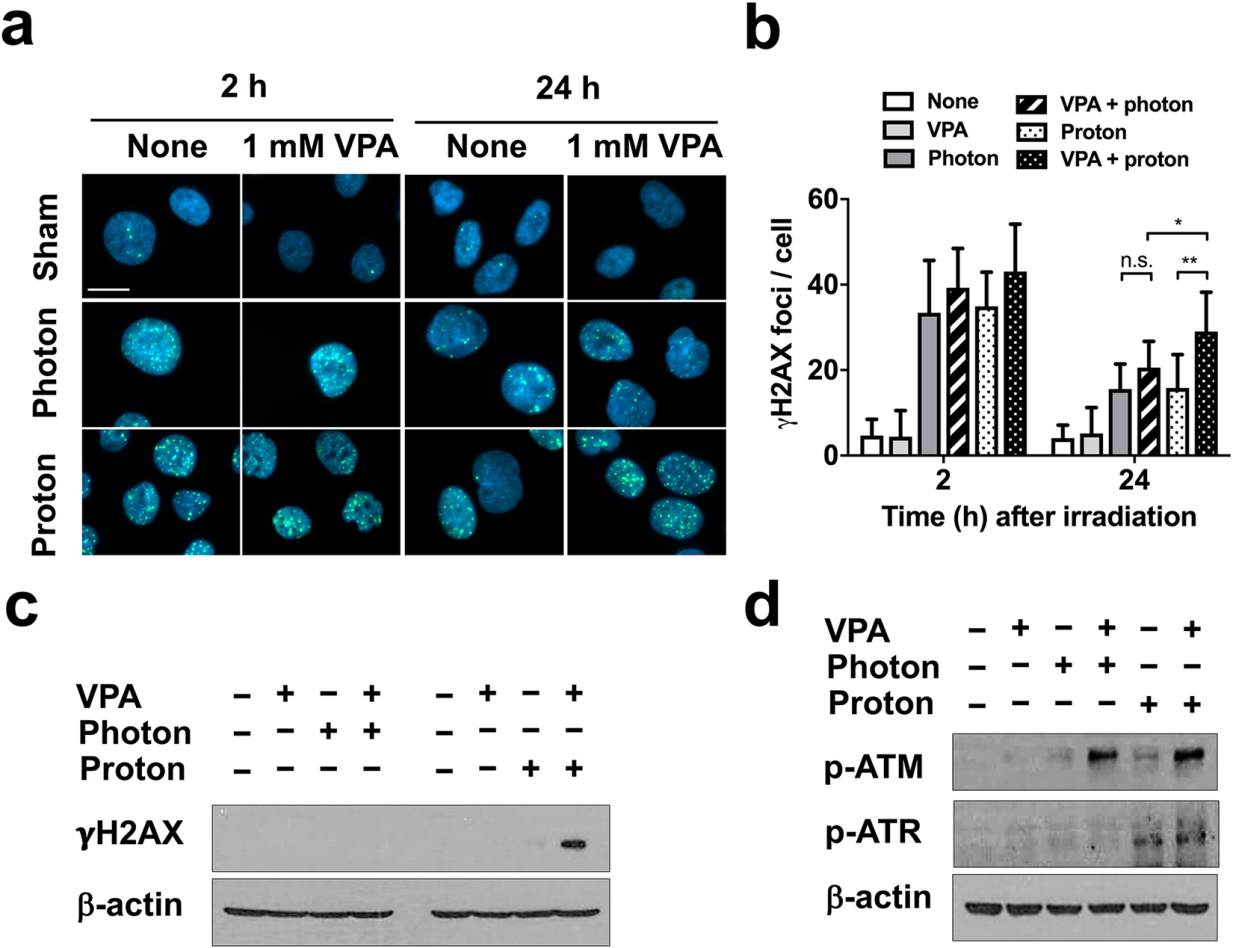
VPA attenuates radiation-induced DNA damage repair in Hep3B cells. (**a**) DNA damage repair after photon or proton irradiation was assessed by γ-H2AX immunostaining. γ-H2AX foci (green) in nuclei (blue) were visualized at 2 h and 24 h after irradiations. Scale bar, 20 µm. (**b**) Quantification of the number of γ-H2AX foci per cell. Data are mean values ± SD of twelve cells. n.s., not significant; *p < 0.05; **p < 0.01. (**c**) Western blot analysis reveals combined treatment with proton and VPA led to delayed abrogation of γ-H2AX. (**d**) Combined treatment with proton and VPA led to persistent activation of ATM and ATR. The samples were harvested 72 h after 6 Gy irradiation. β-actin is used as a loading control. The cropped blots are presented and their full-length blots are included in the Supplementary Fig. S1.

To test whether VPA affects radiation-induced DNA damage repair, we monitored formation of γ-H2AX foci, a sensitive biomarker of DNA double strand breaks. Immunofluorescence analysis showed that γ-H2AX foci formation prominently increased with either photon or proton irradiation at 2 h and then decreased at 24 h (Fig. 4a and b). VPA treatment alone did not have an impact on γ-H2AX foci formation in non-irradiated cells but it markedly attenuated resolution of radiation-induced γ-H2AX foci (Fig. 4a and b), which was more evident in proton-irradiated cells (*p* < 0.01).

Effect of VPA in DNA damage response signaling was determined by western blot analysis of γ-H2AX, phosphorylated ATM and ATR. γ-H2AX expression disappeared at 72 h of post-irradiation but it remained persistent in cells co-treated with VPA and proton beam irradiation (Fig. 4c). Phosphorylation of ATM and ATR was slightly higher in proton-irradiated cells and strongly enhanced by VPA co-treatment at 72 h of post-irradiation (Fig. 4d).

### VPA enhances proton irradiation-induced apoptosis

**Figure 5:**
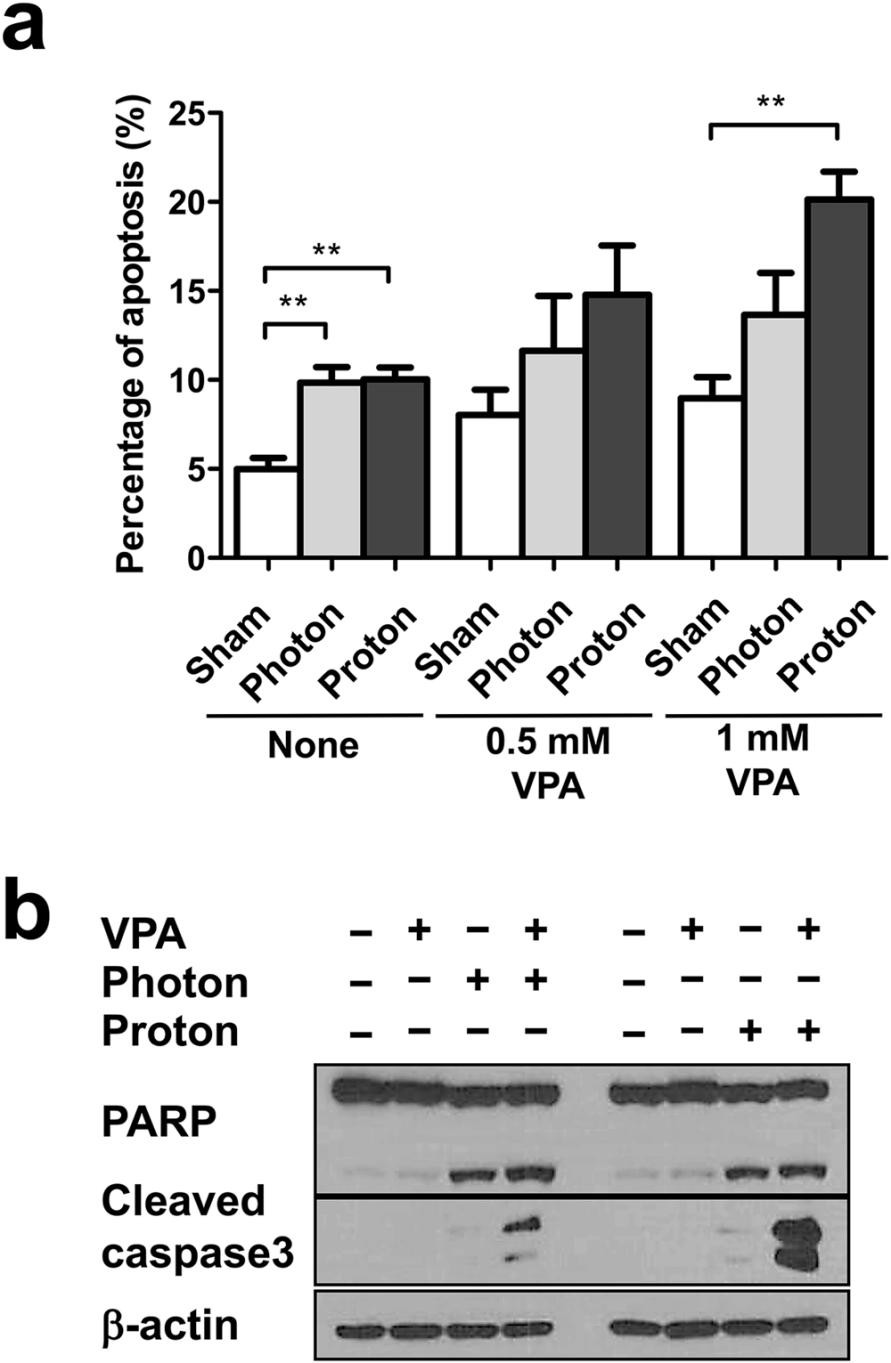
VPA enhances proton-induced apoptosis in Hep3B cells. (**a**) Population of apoptotic cells at 72 h after radiation treatment was assessed by flow cytometry using Annexin-V staining. Data are mean values ± SD of three samples **p < 0.01. (**b**) Western blot analysis of cleaved PARP and caspase-3 antibodies showed VPA augmented proton-induced apoptosis. The cropped blots are presented and their full-length blots are included in the Supplementary Fig. S1.

Next, we determined effect of VPA on radiation-induced apoptosis using flow cytometry. Irradiation of photon and proton beam increased apoptotic cell population from 5.0% to 9.9% and 10.0%, respectively (Fig. 5a). The co-treatment with VPA further increased the proportion of apoptosis: combination of proton and 1 mM VPA (20.1%) induced more apoptotic cell death than that of photon and 1 mM VPA (12.7%).

The increment of apoptosis by combination treatment with proton irradiation and VPA was further confirmed by western blot analysis (Fig. 5b). The cleavage of PARP was induced by either photon or proton irradiation, which was further enhanced by VPA treatment. Cleaved caspase-3 level was the most prominent in the Hep3B cells co-treated with VPA and proton irradiation.

### VPA enhances proton-induced accumulation of reactive oxygen species (ROS)

**Figure 6:**
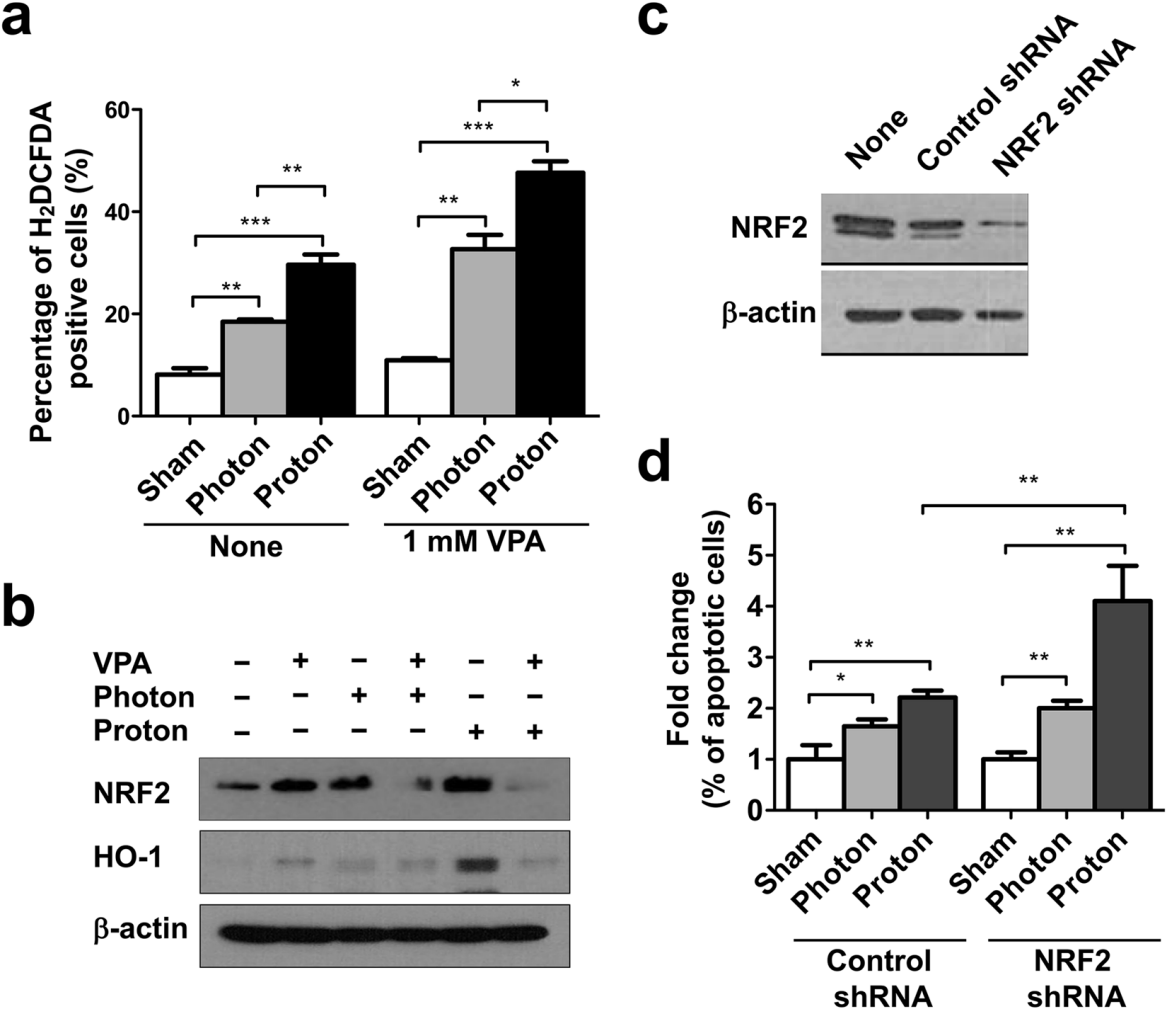
VPA enhances proton-induced ROS production and suppresses activation of NRF2 signaling in Hep3B cells. (**a**) Production of ROS in 6 Gy photon or proton-irradiated cells was measured using H2DCADA fluorescence dye. Data are mean values ± SD of three samples. *p < 0.05, **p < 0.01, ***p < 0.001. (**b**) Western blot analysis showed VPA suppressed proton-mediated upregulation of NRF-2 and its downstream HO-1. (**c**) Western blot analysis confirmed shRNA-mediated depletion of NRF2 in Hep3B. GFP shRNA was used as a control shRNA. (**d**) Apoptosis assay using flow cytometry with annexin V revealed proton irradiation induced more apoptosis of NRF2-depleted cells than control cells. Data are mean values ± SD of three samples. *p < 0.05, **p < 0.01, ***p < 0.001. The cropped blots are presented and their full-length blots are included in the Supplementary Fig. S1.

Since radiation-induced DNA damage is mainly due to ROS generation following water radiolysis, we determined the effect of VPA on radiation-induced accumulation of ROS level in Hep3B cells. The ROS sensitive fluorescent dye, 2′, 7′-dichlorodihydrofluorescein diacetate (H2DCF-DA) was used for ROS detection. Flow cytometry analysis showed that irradiation increased intracellular ROS level and the percentage of H2DCF-DA positive cells was even higher upon proton than photon irradiation (Fig. 6a). The percentage of H2DCF-DA positive cells were further increased by the addition of 1 mM VPA, suggesting that VPA enhanced proton-induced ROS production.

Next, we tested effect of VPA on signaling of nuclear factor erythroid 2-related factor 2 (NRF2), a key regulator of cellular redox homeostasis. Western blot analysis of NRF2 showed irradiation increased NRF2 expression as seen in previous reports (Fig. 6b). Interestingly, NRF2 expression was more induced by proton irradiation than photon irradiation, which would be due to more ROS production upon proton irradiation than photon irradiation. Expression of heme oxygenase 1, HO-1, a target gene of NRF2, was dramatically increased by proton irradiation. However, VPA treatment abrogated the proton-induced expressions of both NRF2 and HO-1 (Fig. 6b). To test whether NRF2 level is related to sensitivity to proton irradiation, we depleted NRF2 in Hep3B by shRNA transfection (Fig. 6c). The increment of apoptotic cell population in NRF2-depleted cells was seen upon proton irradiation compared to photon irradiation (*p* < 0.01) and control shRNA cells (*p* < 0.01), suggesting that NRF2 may protect cells from proton-induced cell killing (Fig. 6d).

### VPA enhances proton-mediated suppression of Hep3B xenograft tumor growth

**Figure 7:**
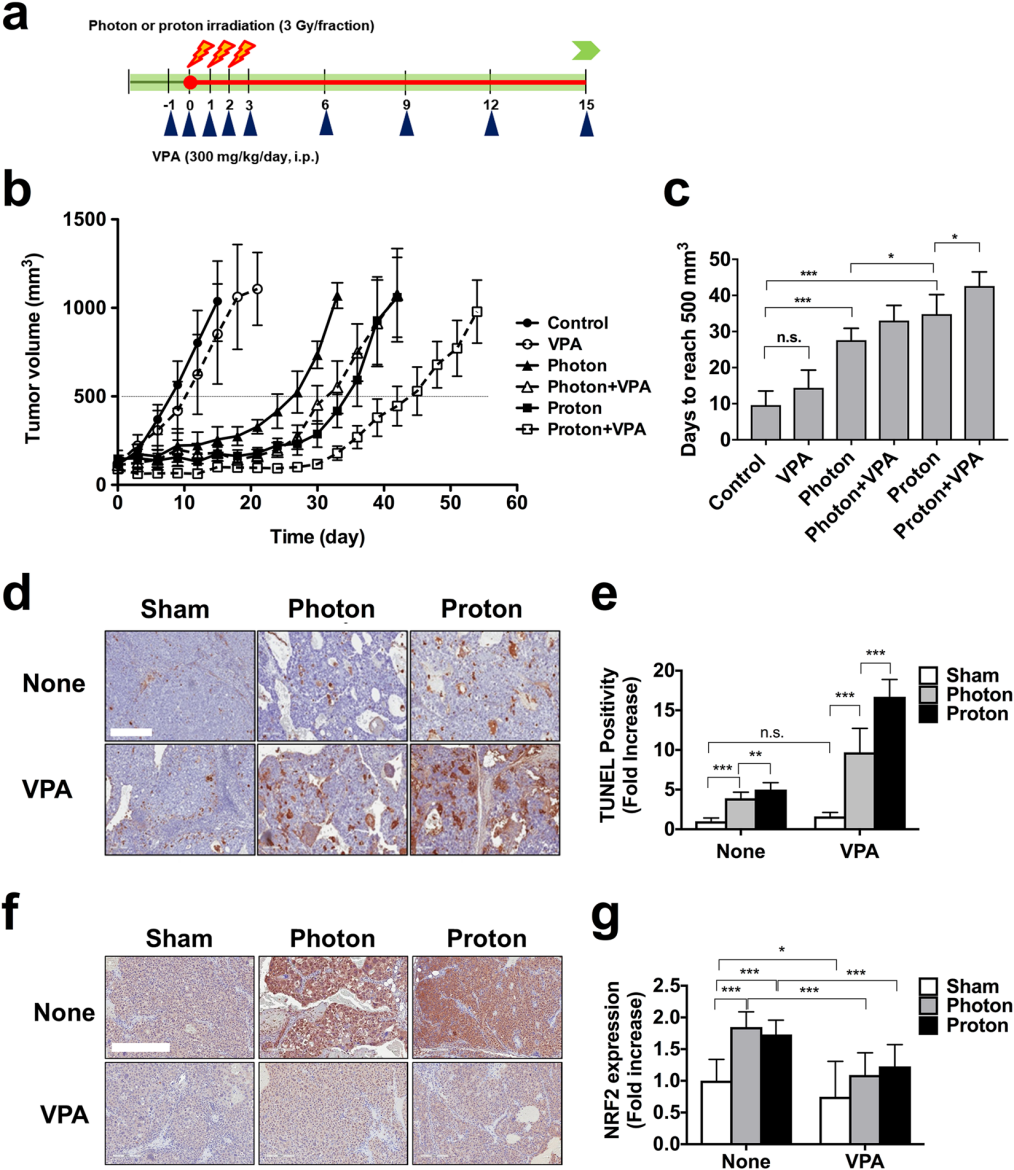
VPA enhances proton-induced tumor growth delay in a Hep3B xenograft model. (**a**) Schematic diagram of the experimental procedure. (**b**) Administration of VPA further suppressed growth of proton-irradiated tumors. Hep3B cells were implanted into right legs of BALB/c nude mice. Once tumors were palpable, they were irradiated with 3 Gy for 3 consecutive days for a total 9 Gy. Mice were treated with intraperitoneal injections of VPA (300 mg/kg/day) every 3 days. Shown are mean tumor volumes and standard deviation per group (n = 4). (**c**) Tumour growth delay was determined by calculating days each tumour taken to reach 500 mm^3^. n.s. not significant; *p < 0.05; ***p < 0.001. (**d**) TUNEL assay detected more apoptotic cells in tissues co-treated with proton and VPA compared to proton alone or combined photon and VPA. Little was seen in tissues treated with VPA alone. Scale bar, 400 µm. (**e**) Quantification of the TUNEL positive cell density in tumor tissue sections. n.s. not significant; **p < 0.01; ***p < 0.001. (**f**) Proton and photon irradiations increased NRF2 expression on tumour tissues, which was suppressed by co-treatment with VPA. NRF2 expression was assessed by immunohistochemistry. Scale bar, 400 µm. (**g**) Quantification of NRF2 expression in tissue samples. *p < 0.05; ***p < 0.001.

We evaluated the *in vivo* efficacy of VPA on proton radiosensitization using Hep3B mouse xenograft model. Nude mice implanted with Hep3B cells were treated with PBS (control), VPA (300 mg/kg, every three days), photon irradiation alone (three daily fractions of 3 Gy), photon irradiation and VPA, proton irradiation alone (three daily fractions of 3 Gy) or proton irradiation and VPA (Fig. 7a).

The Hep3B tumor growth of each treatment group was compared on day 15. Photon (256.1 ± 161.5, *p* < 0.05) and proton irradiation (175.5 ± 93.1, *p* < 0.01) significantly inhibited Hep3B tumor growth, compared to control group (1,038.1 ± 555.4 mm^3^) (Fig. 7b). VPA alone did not inhibit tumor growth (852.2 ± 555.4 mm^3^, *p* = 0.619). No significant difference between proton irradiation and photon irradiation group was seen (*p* = 0.326). To clarify effect of VPA on tumor growth, we calculated tumor growth delay (TGD) that is defined as a difference in the time to reach tumor volume of 500 mm^3^ between control group and experimental group (Fig. 7c). Either photon or proton irradiation significantly delayed tumor growth (*p* < 0.001); the TGDs of photon irradiation and proton irradiation groups relative to control group were 18.0 days and 25.2 days, respectively and proton was more effective than photon (*p* < 0.05). VPA alone did not affect tumor growth (TGD of 4.8 days, *p* = 0.126) compared to control group. However, VPA further attenuated tumor growth of mice in the photon irradiation group (TGD of 5.4 days, *p* = 0.05) and it significantly delayed tumor growth of mice in the proton irradiation group (TGD of 7.8 days, *p* < 0.05).

In accordance with *in vitro* data, TUNEL assay on tissue sections from the transplanted tumours revealed that both irradiations increased apoptotic cells *in vivo*, which were further enhanced by VPA treatment (Fig. 7d). Quantification data confirmed that VPA alone did not affect apoptosis on the tissues but its combined treatment with either photons or protons led to a significant enhancement of apoptosis in the tumour tissues (*p* < 0.001, Fig. 7e). In addition, immunohistochemical analysis on the tumour tissues showed that NRF2 expression was greatly induced by both irradiations (*p* < 0.001, Fig. 7f), which is well consistent with *in vitro* results (Fig. 6b). VPA treatment suppressed radiation-induced NRF2 expression (*p* < 0.001). Collectively, our data suggest that VPA exerted *in vivo* radiosensitizing effect via induction of apoptosis and suppression of NRF2.

## Discussion

In this study, the radiosensitizing effect of a HDAC inhibitor, VPA on human HCC cells was evaluated with two different types of radiation, photon and proton using *in vitro* and *in vivo* models. To the best of our knowledge, this is the first study to directly compare the combined effect of a HDAC inhibitor on photon and proton irradiations. It is intriguing that VPA exerted a stronger sensitizing effect when combined with proton irradiation, compared to photon irradiation. Enhanced DNA damages and accumulated ROS production were seen when VPA and proton irradiation was co-treated. Consistent with *in vitro* data, VPA enhanced proton-mediated suppression of xenograft tumor growth *in vivo*, suggesting that VPA could be a valuable proton sensitizer in HCC treatments.

The RBE of proton irradiation is not an issue because it is well recognized, studied, and actively adapted in oncologic fields^21^. It is demonstrated that proton RBE can be affected by initial proton beam energy, linear energy transfer, dose per fraction, cell types, and proton therapy machines. Regardless of the complexity and/or uncertainty, “generic RBE” which means a single all-round value of RBE 1.1 is widely used in most clinical proton therapy centers. Radiosensitizing effect of chemotherapy drugs like cisplatin, 5-fluorouracil and capecitabine on conventional photon therapy has been well studied and is simply being applied to proton treatment in a clinical setting^29–31^. The use of proton beam therapy and capecitabine for patients with resectable pancreatic ductal adenocarcinoma is well tolerated with favorable local control^29^. In an esophageal cancer study, proton beam therapy combined with cisplatin and 5-fluorouracil seemed to be feasible with regard to late cardio-pulmonary toxicity^30^. In addition, high dose proton beam therapy with cisplatin and vinorelbine is safe to use for treatment of unresectable stage III non-small cell lung cancer^31^. Considering differences in biological effects between proton and photon irradiations, there is, however, no doubt that proton RBE could be affected by other chemotherapeutic agents. This prompted us to investigate an effect of HDAC inhibitors that are recently highlighted as a radiosensitizer, on proton RBE.

In addition to their potential as anticancer therapeutics^10^, HDAC inhibitors have been tested as radiosensitizer. For example, suberoylanilide hydroxamic acid (SAHA, vorinostat) enhanced photon irradiation-mediated cell killing by inhibiting DNA double strand break repair^17^. SAHA inhibited radiation-induced upregulation of RAD50 and RAD51 in diverse types of cancer cells, suggesting a possible role of impaired homologous recombination (HR) repair pathway in SAHA-induced radiosensitization^14,16,17^. SAHA-mediated downregulation of Ku70 and Ku80 protein was seen in colon cancer cells, suggesting implication of non-homologous end-joining (NHEJ) DNA repair pathway in the radiosensitization^15^. As with SAHA, increased efficacy by combination treatment with VPA and photon irradiation has been reported in preclinical and/or clinical setting^25,26,32^. In consistent with the previous results, enhanced radiation sensitivity by VPA was also seen in our study with HCC cells. The sensitizing effect of VPA was higher in proton-irradiated cells than photon-irradiated cells; VPA enhanced proton-induced apoptosis both *in vitro* and *in vivo*. Our findings indicate that stronger sensitizing effect of VPA in response to proton over photon irradiation increased proton RBE, which may allow to de-escalate prescription doses and protect normal tissues in a clinical setting.

Recent preclinical studies have shown that HR repair pathway is more necessary for repair of proton-induced DNA damages than NHEJ^33–35^. Defects in HR pathway increased proton RBE in lung cancer cells^34^ and SAHA-mediated RAD51 downregulation enhanced proton sensitizing effect in lung adenocarcinoma A549 cells^33^. In our study with HCC cells, altered expression of HR pathway proteins was not seen in VPA-treated cells. Instead, co-treatment with VPA increased proton-induced G2/M arrest after 72 h and attenuated proton-induced DNA damage repair, which was judged by persistence of γH2AX foci and prolonged activation of ATM/ATR. Thus, this suggested implication of a different mechanism rather than suppression of expression of DNA damage repair genes in VPA-mediated proton radiosensitization.

The DNA damages induced by sparsely ionizing radiations, like photon and proton beam is mainly caused by ROS produced during water radiolysis. In our study, more accumulation of ROS was seen in the Hep3B cells treated with proton than photon, which is consistent with previous studies^36,37^. Level of NRF2, a key transcription factor protecting from oxidative stress, increased concomitantly with the increase in ROS level after irradiations, which was prominent in proton-irradiated cells. VPA alone also slightly increased NRF2 level, which might be related to increased ROS level. It has been shown that VPA^38,39^ and other HDAC inhibitors^40–43^ increased intracellular ROS levels through NADPH oxidase activation. Even though ROS levels were further increased by co-treatment with radiation and VPA, proton-induced NRF2 expression was suppressed by VPA treatment. Moreover, VPA-mediated suppression of expression of HO-1, a target gene of NRF2, suggested that VPA inhibited radiation-induced activation of NRF2 signaling. Suppression of NRF2 expression by VPA^44–46^ and other HDAC inhibitors^47,48^ were also seen in cancer and other disease and is proposed to be a plausible approach to enhance antitumor activity of TRAIL or EGFR inhibitors. Taken together, our data suggest that VPA suppressed induction of NRF2 in response to proton-mediated ROS overproduction and downregulated expression of anti-oxidative genes such as HO-1, thereby resulting in enhancement of RT-induced apoptosis and reduction in clonogenic survival.

NRF2 is frequently activated in HCC (5–15%) and is considered as one of oncogenic drivers including TERT and β-catenin^49,50^. Activated NRF2 confers radioresistance and chemoresistance by protecting cells from radiation-induced oxidative stress and detoxifying broad spectrum of anticancer drugs. In a non-small lung cancer, genetic modulation of NRF2 levels affects radiation sensitivity and NRF2 knockdown potentiates radiation-induced apoptosis^51,52^, which is well consistent with our results. Similar findings were also seen in various cancers including prostate^53,54^, squamous cell lung cancer^55^, hepatocellular carcinoma^56^ and glioblastoma cells^57^. A recent study on squamous cell lung cancer patients revealed that NRF2 status is a predictive marker of local recurrence after radiation therapy^58^. As our data clearly showed that depletion of NRF2 by VPA treatment or shRNA transfection made cancer cells much more sensitive to proton irradiation, targeting NRF2 pathway may impact outcome of proton radiation therapy.

This preclinical study clearly showed a reliable combined effect of VPA with proton irradiation in HCC *in vitro* and *in vivo*, but there remain several obstacles for clinical use of VPA upon proton therapy for HCC treatment. Whereas the optimum dose of VPA as antiepileptic drug is well documented, its optimal dose for radiosensitization is still under investigation^20^. Based on the previous preclinical studies^25,27,59^, we used 300 mg/kg/day for our *in vivo* study, but it is higher than the maximum daily recommended dose (60 mg/kg/day) used for epilepsy. Furthermore, hepatotoxicity would be an issue of VPA treatment in HCC patients^60^. Saha *et al*. showed that valproic acid dose-dependently inhibited viability of human HCC cells such as HepG2 and SNU475 but not normal hepatocyte MIHA cells^61^. Another study showed that VPA radiosensitized glioblastoma cells but not normal hippocampal neurons, suggesting the effect of VPA may be limited to malignant cells^25^. Maintenance of liver function is important for treatment of HCC because HCC is mostly developed in liver cirrhosis background. Thus, more comprehensive dose optimization of VPA for radiosensitization will be needed before clinical use for HCC patients.

## Conclusion

Our study highlights a radiosensitizing effect of VPA, particularly with proton radiation in HCC treatment *in vitro* and *in vivo*. VPA potentiated the effect of photon or proton irradiation in HCC cells, resulting in an increase in proton RBE. As proton is only 10% more effective than photon, it should be interesting to address effect of VPA on carbon beam irradiation which has higher RBE. NRF2 is an emerging prognostic marker of HCC and other cancer types and contributes to radioresistance. Thus, targeting NRF2 with VPA may be a promising strategy for cancer patients who receive proton radiation therapy.

## Materials and Methods

### Cell culture

The HCC cell lines, Hep3B and Huh7 were purchased from Korean Cell Line Bank and were cultured in DMEM medium supplemented with 10% fetal bovine serum and 1x Antibiotic-Antimycotic (Gibco, Carlsbed, CA, USA). All cultures were maintained at a humidified 37 °C incubator with 5% CO_2_ atmosphere and were routinely passaged every 2–3 days.

### Reagents and antibodies

VPA was purchased from Sigma-Aldrich (St. Louse, MO, USA). Carboxy-H2DCFDA was purchased from Thermo Fisher Scientific (Waltham, MA, USA). FITC annexin V apoptosis detection kit was purchased from BD biosciences (San Diego, CA, USA). Polyclonal antibody specific for acetyl-histone H4 were purchased from Millipore (Billerica, MA, USA). Polyclonal antibody specific for NRF2 was purchased from Santa Cruz Biotechnology (Santa Cruz, CA, USA). Antibodies specific for cleaved caspase-3, cleaved PARP, HO-1, and phospho-H2AX (Ser139) were purchased from Cell Signaling Technology (Danvers, MA, USA). Anti-actin antibody was purchased from Sigma-Aldrich.

### Irradiation of photon and proton

Cells were seeded into a 6-well plate or a 10-cm culture plate and then were irradiated with either photon or proton beam next day. Photon beam irradiation was performed with a linear accelerator Varian Clinac 6EX machine (Varian Medical Systems, Palo Alto, CA, USA) at Samsung Medical Center. The cell dishes were placed under 2 cm thickness solid water phantom with source surface distance of 100 cm and field size of 30 × 30 cm and were irradiated with 6-MV photons at a dose rate of 3.96 Gy per min. The photon absolute dose was calibrated according to TG-51 and verified with Gafchromic film to 1% accuracy.

Proton beam irradiation was performed with a proton therapy machine of Samsung Proton Therapy Center in Seoul, South Korea^11^. Cells were irradiated with 230 MeV proton beam with wobbling method^62^ and the field was collimated by 18 × 12 cm Brass block. The range of proton beam was 22.8 cm (distal 90%) and the spread-out Bragg peak (SOBP) width was 11.2 cm (distal 90% to proximal 95%). The irradiation point of cell dishes was the middle of SOBP (17.2 cm) and a dose rate of 2.14 Gy per min. To minimize the dose uncertainty, the absolute dose was verified according to TRS-398 for proton therapy to 1% accuracy. The graded doses of 2, 4, 6 and 10 Gy were delivered by application of anterior beams of photon or proton irradiation.

### Cell proliferation assay

Colorimetric MTT (thiazolyl blue tetrazolium bromide) metabolic activity assay was used to determine proliferation of cells treated with VPA. Cells were seeded at 1 × 10^3^ cells/well into a 96-well plate and on the next day the media were replaced with fresh media containing 0, 0.25, 0.5, 1, 2 and 5 mM VPA. After 48 h incubation, MTT solution (0.5 mg/ml) was added and population of metabolically active cells was calculated based on formazan formation, which was monitored by measuring the absorbance at 540 nm using SpectraMax i3 microplate reader (Molecular Devices, Sunnyvale, CA, USA). The cell proliferation was calculated as percentage of untreated control.

### Clonogenic assay

Cells were seeded into a 6-well plate in triplicate (300 cells/well for 0 and 2 Gy, 600 cells/well for 4 Gy and 1000 cells/well for 6 Gy) and then incubated overnight. The cells were pre-treated with 1 mM VPA for 3 h and were irradiated with either photons or protons of doses of 0, 2, 4 and 6 Gy. After incubating for 14 days, cells were fixed and stained with methanol containing 1% crystal violet and colonies containing 50 or more cells were counted. Plating efficiency was calculated as % of colonies from seeded cells and cell survival at each irradiation dose was determined by dividing the plating efficiency of the irradiated cells by that of the mock-irradiated control. The survival curves were fitted and analysed using linear-quadratic model [SF = exp(−αD − βD^2^)] using GraphPad Prism 7.02 (GraphPad Software, La Jolla, CA, USA); SF is the survival fraction and D is the absorbed dose. Proton RBE was calculated as the ratio of the physical doses of photon radiation and protons that correspond to the survival fraction of 0.1.

### Western blot analysis

Cells irradiated with photon or proton were harvested in a lysis buffer and an equal amount of proteins was subjected to SDS-PAGE. Proteins were transferred onto nitrocellulose membrane and blots were probed with antibodies specific for the indicated proteins. The protein bands were visualized by Amersham enhanced chemiluminescence reaction kit (GE healthcare, Piscataway, NJ, USA) and exposure to X-ray film.

### Immunofluorescence staining

2 × 10^4^ Hep3B cells were seeded on a cover glass (Marinfild Inc., Rochester, NY, USA) a day before irradiation. The cells were pre-treated with VPA for 3 h and then exposed to photon or proton and then fixed with 4% formaldehyde at the indicated times. After permeabilized using 0.01% Triton X-100 and blocked with 2% bovine serum albumin for 30 min, cells were incubated with phospho-S193 H2AX antibody for 2 h, followed by incubating with Alexa Fluor 488-conjugated secondary antibody (Life Technologies, Paisley, UK) and 4′,6-diamidino-2-phenylindole (DAPI) for 30 min at room temperature. After mounting cover glass with SlowFade anti-fade reagent (Molecular Probes, Eugene, OR, USA), immunofluorescent images were acquired using fluorescent microscope (Zeiss Observer D1, Carl Zeiss Co., Ltd., Jena, Germany).

### Reactive oxygen species (ROS) measurement

Cells were pre-treated with 1 mM VPA for 3 h and then incubated with 20 μM Carboxy-H2DCFDA for 30 min, followed by exposure to 6 Gy of photon or proton irradiation. After 72 h post-irradiation, the cells were harvested and ROS level was analysed by flow cytometry (FACSVerse, Becton-Dickinson, CA, USA).

### Cell cycle analysis

Cell cycle analysis was performed by flow cytometry. 2 × 10^5^ cells were plated in 6-well plates and allowed to attach overnight. Then, cells were pre-treated with VPA for 3 h and then exposed to photon or proton. At 48 h of post-irradiation, cells were collected, fixed with pre-chilled 70% ethanol and washed with phosphate-buffered saline (PBS). The cells were resuspended in 1 ml of PBS containing 1 mg/ml RNase and 50 μg/ml propidium iodide, incubated in the dark for 30 min at 37 °C, and analysed by flow cytometry.

### shRNA transfection

Transient transfection with short hairpin RNAs was performed to knock down NRF2 expression. The shRNA plasmid for NRF2 depletion was purchased from Sigma (TRCN0000273494,) and its sequence is: 5′-CCGGAGTTTGGGAGGAGCTATTATCCTCGAGGATAATAGCTCCTCCCAAACTTTTTTG-3′. For transfection, Hep3B cells (1 × 10^5^ cells) were seeded on 6 well plates and incubated with a mixture of 2 $\mu$g of NRF2 shRNAs and 10 $\mu$l of Lipofectamine 3000 (Invitrogen, Carlsbad, CA, USA) for 4 h and then the media was replaced with fresh one. Depletion of NRF2 in Hep3B was confirmed by western blot analysis using anti-NRF2 antibody.

### Animal experiments

Six to seven-week-old female Balb/c nude mice were purchased from Orient Bio (Gapyeong, South Korea). 5 × 10^6^ Hep3B cells were injected subcutaneously into the right hind leg. When the mean tumor volume reached 50~100 mm^3^, mice were randomized to six groups (n = 6) to receive the following treatments: (a) PBS, 0.1 mL, (b) VPA, (c) photon irradiation, (d) VPA + photon irradiation, (e) proton irradiation and (f) VPA + proton irradiation. Mice were pre-treated with intraperitoneal injections of VPA (300 mg/kg/day) for 4 days. 300 mg/kg/day of VPA is within an acceptable range to administrate in mice^25,59^ and no body weight change was seen in mice. From the 2nd day of drug treatment, the tumor-bearing right hind legs were irradiated with photons or protons at a dose of 3 Gy per fraction to a total dose of 9 Gy for 3 consecutive days. After irradiation, injection of VPA continued every 3 days until the day before sacrifice to keep the effect of VPA active in the mice. Tumor volumes were measured every 3 days with calipers and calculated according to following formula: volume = *L* × (*W*)^2^ × 1/2 (*L*, length in mm; *W*, width in mm)^27,63,64^. The animal experiments were reviewed and approved by the Institutional Animal Care and Use Committee (IACUC) of Samsung Biomedical Research Institute, which is accredited by an Association for Assessment and Accreditation of Laboratory Animal Care International (AAALAC, protocol number H-A9–003). All experiments were performed in accordance with relevant guidelines and regulations.

### TUNEL assays

Terminal deoxynucleotidyl transferase dUTP nick end labeling (TUNEL) assay was performed to detect apoptosis in formalin-fixed paraffin-embedded (FFPE) tissues. Irradiated tumor tissues were fixed with 10% neutral buffered formalin (NBF) for 4 h and embedded in paraffin. After deparaffinization, TUNEL staining was performed using *In Situ* Cell Death Detection Kit (Roche Diagnostics, Mannheim, Germany). Images were captured using an Aperio ScanScope AT slide scanner (Leica Biosystems Inc. Buffalo Grove, Illinois, USA) and analysed using ImageScope software (Leica Biosystems).

### Immunohistochemistry

To evaluate expression of NRF2 in tumour tissues, immunohistochemistry (IHC) was performed. The sections sliced into 4 µm were deparaffinized in xylene, rehydrated in graded alcohol, and transferred to 0.01 M PBS, pH 7.4. After heat induced epitope retrieval (HIER) with citrate buffer (pH 6.0; Dako, Carpinteria, CA) for 3 min at 121 °C to reveal hidden antigen epitopes, endogenous peroxidase was blocked with 3% hydrogen peroxide in PBS for 10 min at room temperature. After washing in PBS buffer, sections were treated with serum free blocking solution (Dako) for 20 minutes at room temperature to block nonspecific binding. Subsequently, sections were incubated with anti-Nrf2 rabbit polyclonal antibody (1/100; Abcam, Cambridge, UK) overnight at 4 °C. After washing in PBS, the sections were incubated for 30 minutes at room temperature with HRP-labelled polymer conjugated secondary antibodies against mouse IgG (Dako) or rabbit IgG (Dako). The colour reaction was developed using the ready-to-use DAB (3,3′-diaminobenzidine) substrate-chromogen solution (Dako) for 5 minutes and then washed with distilled water. Finally, sections were lightly counterstained with Mayer’s haematoxylin for 30 seconds before dehydration and mounting. Slides were scanned with Aperio ScanScope AT slide scanner (Leica Biosystems Inc. Buffalo Grove, Illinois, USA) at 20× magnification and analysed using ImageScope software (Leica Biosystems). Pixel counts were gated to strongly positive pixel counts using the Positive Pixel Count v9 (PPCv9) algorithm embedded in the program.

### Statistical analysis

All data was expressed as the mean ± SD from at least three independent experiments. Statistical analysis was performed using GraphPad Prism 7.02. Statistical significance was determined by unpaired, two-tailed Student’s *t*-test and *p* < 0.05 was considered statistically significant.

## Electronic supplementary material

Supplementary information

## References

[1] Wagner JM, Hackanson B, Lubbert M, Jung M (2010). Histone deacetylase (HDAC) inhibitors in recent clinical trials for cancer therapy. Clin Epigenetics.

[2] Gray SG, Ekstrom TJ (2001). The human histone deacetylase family. Exp Cell Res.

[3] Strahl BD, Allis CD (2000). The language of covalent histone modifications. Nature.

[4] Groselj B, Sharma NL, Hamdy FC, Kerr M, Kiltie AE (2013). Histone deacetylase inhibitors as radiosensitisers: effects on DNA damage signalling and repair. Br J Cancer.

[5] West AC, Johnstone RW (2014). New and emerging HDAC inhibitors for cancer treatment. J Clin Invest.

[6] Sharma S, Kelly TK, Jones PA (2010). Epigenetics in cancer. Carcinogenesis.

[7] Glozak MA, Sengupta N, Zhang X, Seto E (2005). Acetylation and deacetylation of non-histone proteins. Gene.

[8] Santoro F (2013). A dual role for Hdac1: oncosuppressor in tumorigenesis, oncogene in tumor maintenance. Blood.

[9] Wilson AJ (2006). Histone deacetylase 3 (HDAC3) and other class I HDACs regulate colon cell maturation and p21 expression and are deregulated in human colon cancer. J Biol Chem.

[10] Lane AA, Chabner BA (2009). Histone deacetylase inhibitors in cancer therapy. J Clin Oncol.

[11] Chung K (2015). The first private-hospital based proton therapy center in Korea; status of the Proton Therapy Center at Samsung Medical Center. Radiat Oncol J.

[12] Kamada T (2015). Carbon ion radiotherapy in Japan: an assessment of 20 years of clinical experience. Lancet Oncol.

[13] Kang JK (2016). The clinical utilization of radiation therapy in Korea between 2009 and 2013. Radiat Oncol J.

[14] Blattmann C (2010). Enhancement of radiation response in osteosarcoma and rhabdomyosarcoma cell lines by histone deacetylase inhibition. Int J Radiat Oncol Biol Phys.

[15] Chen X, Wong P, Radany E, Wong JY (2009). HDAC inhibitor, valproic acid, induces p53-dependent radiosensitization of colon cancer cells. Cancer Biother Radiopharm.

[16] Chinnaiyan P, Vallabhaneni G, Armstrong E, Huang SM, Harari PM (2005). Modulation of radiation response by histone deacetylase inhibition. Int J Radiat Oncol Biol Phys.

[17] Munshi A (2006). Vorinostat, a histone deacetylase inhibitor, enhances the response of human tumor cells to ionizing radiation through prolongation of gamma-H2AX foci. Mol Cancer Ther.

[18] Ree AH (2010). Vorinostat, a histone deacetylase inhibitor, combined with pelvic palliative radiotherapy for gastrointestinal carcinoma: the Pelvic Radiation and Vorinostat (PRAVO) phase 1 study. Lancet Oncol.

[19] Barker CA, Bishop AJ, Chang M, Beal K, Chan TA (2013). Valproic acid use during radiation therapy for glioblastoma associated with improved survival. Int J Radiat Oncol Biol Phys.

[20] Krauze AV (2015). A Phase 2 Study of Concurrent Radiation Therapy, Temozolomide, and the Histone Deacetylase Inhibitor Valproic Acid for Patients With Glioblastoma. Int J Radiat Oncol Biol Phys.

[21] Paganetti H (2014). Relative biological effectiveness (RBE) values for proton beam therapy. Variations as a function of biological endpoint, dose, and linear energy transfer. Phys Med Biol.

[22] Dagrosa MA (2011). First evaluation of the biologic effectiveness factors of boron neutron capture therapy (BNCT) in a human colon carcinoma cell line. Int J Radiat Oncol Biol Phys.

[23] Redjal N (2016). Valproic acid, compared to other antiepileptic drugs, is associated with improved overall and progression-free survival in glioblastoma but worse outcome in grade II/III gliomas treated with temozolomide. J Neurooncol.

[24] Zhou Y (2014). Histone deacetylase inhibitor, valproic acid, radiosensitizes the C6 glioma cell line *in vitro*. Oncol Lett.

[25] Thotala D (2015). Valproic acid enhances the efficacy of radiation therapy by protecting normal hippocampal neurons and sensitizing malignant glioblastoma cells. Oncotarget.

[26] Makita N (2015). Inhibitory effects of valproic acid in DNA double-strand break repair after irradiation in esophageal squamous carcinoma cells. Oncol Rep.

[27] Chie EK (2015). *In Vitro* and *In Vivo* Radiosensitizing Effect of Valproic Acid on Fractionated Irradiation. Cancer Res Treat.

[28] Chiou HY (2015). Valproic acid promotes radiosensitization in meningioma stem-like cells. Oncotarget.

[29] Hong TS (2014). A phase 1/2 and biomarker study of preoperative short course chemoradiation with proton beam therapy and capecitabine followed by early surgery for resectable pancreatic ductal adenocarcinoma. Int J Radiat Oncol Biol Phys.

[30] Ishikawa H (2015). Proton beam therapy combined with concurrent chemotherapy for esophageal cancer. Anticancer Res.

[31] Oshiro Y (2014). High-dose concurrent chemo-proton therapy for Stage III NSCLC: preliminary results of a Phase II study. J Radiat Res.

[32] Reddy JP (2015). Antiepileptic drug use improves overall survival in breast cancer patients with brain metastases in the setting of whole brain radiotherapy. Radiother Oncol.

[33] Fontana AO (2015). Differential DNA repair pathway choice in cancer cells after proton- and photon-irradiation. Radiother Oncol.

[34] Liu Q (2015). Lung cancer cell line screen links fanconi anemia/BRCA pathway defects to increased relative biological effectiveness of proton radiation. Int J Radiat Oncol Biol Phys.

[35] Grosse N (2014). Deficiency in homologous recombination renders Mammalian cells more sensitive to proton versus photon irradiation. Int J Radiat Oncol Biol Phys.

[36] Di Pietro C (2006). Cellular and molecular effects of protons: apoptosis induction and potential implications for cancer therapy. Apoptosis.

[37] Alan Mitteer R (2015). Proton beam radiation induces DNA damage and cell apoptosis in glioma stem cells through reactive oxygen species. Sci Rep.

[38] Kawai Y, Arinze IJ (2006). Valproic acid-induced gene expression through production of reactive oxygen species. Cancer Res.

[39] Defoort EN, Kim PM, Winn LM (2006). Valproic acid increases conservative homologous recombination frequency and reactive oxygen species formation: a potential mechanism for valproic acid-induced neural tube defects. Mol Pharmacol.

[40] Ruefli AA (2001). The histone deacetylase inhibitor and chemotherapeutic agent suberoylanilide hydroxamic acid (SAHA) induces a cell-death pathway characterized by cleavage of Bid and production of reactive oxygen species. Proc Natl Acad Sci USA.

[41] Rosato RR, Almenara JA, Grant S (2003). The histone deacetylase inhibitor MS-275 promotes differentiation or apoptosis in human leukemia cells through a process regulated by generation of reactive oxygen species and induction of p21CIP1/WAF1 1. Cancer Res.

[42] Rahmani M (2005). Coadministration of histone deacetylase inhibitors and perifosine synergistically induces apoptosis in human leukemia cells through Akt and ERK1/2 inactivation and the generation of ceramide and reactive oxygen species. Cancer Res.

[43] Hu Y (2010). Overcoming resistance to histone deacetylase inhibitors in human leukemia with the redox modulating compound beta-phenylethyl isothiocyanate. Blood.

[44] Cha HY (2016). Downregulation of Nrf2 by the combination of TRAIL and Valproic acid induces apoptotic cell death of TRAIL-resistant papillary thyroid cancer cells via suppression of Bcl-xL. Cancer Lett.

[45] Jin J (2014). Role of Nrf2 activation and NF-kappaB inhibition in valproic acid induced hepatotoxicity and in diammonium glycyrrhizinate induced protection in mice. Food Chem Toxicol.

[46] Palsamy P, Bidasee KR, Shinohara T (2014). Valproic acid suppresses Nrf2/Keap1 dependent antioxidant protection through induction of endoplasmic reticulum stress and Keap1 promoter DNA demethylation in human lens epithelial cells. Exp Eye Res.

[47] Mercado N (2011). Decreased histone deacetylase 2 impairs Nrf2 activation by oxidative stress. Biochem Biophys Res Commun.

[48] Leone A (2015). Vorinostat synergizes with EGFR inhibitors in NSCLC cells by increasing ROS via up-regulation of the major mitochondrial porin VDAC1 and modulation of the c-Myc-NRF2-KEAP1 pathway. Free Radic Biol Med.

[49] Zucman-Rossi J, Villanueva A, Nault JC, Llovet JM (2015). Genetic Landscape and Biomarkers of Hepatocellular Carcinoma. Gastroenterology.

[50] Shibata T, Aburatani H (2014). Exploration of liver cancer genomes. Nat Rev Gastroenterol Hepatol.

[51] Zhao Q (2016). Downregulation of Nrf2 promotes radiation-induced apoptosis through Nrf2 mediated Notch signaling in non-small cell lung cancer cells. Int J Oncol.

[52] Singh A, Bodas M, Wakabayashi N, Bunz F, Biswal S (2010). Gain of Nrf2 function in non-small-cell lung cancer cells confers radioresistance. Antioxid Redox Signal.

[53] Zhang P (2010). Loss of Kelch-like ECH-associated protein 1 function in prostate cancer cells causes chemoresistance and radioresistance and promotes tumor growth. Mol Cancer Ther.

[54] Jayakumar S, Kunwar A, Sandur SK, Pandey BN, Chaubey RC (2014). Differential response of DU145 and PC3 prostate cancer cells to ionizing radiation: role of reactive oxygen species, GSH and Nrf2 in radiosensitivity. Biochim Biophys Acta.

[55] Abazeed ME (2013). Integrative radiogenomic profiling of squamous cell lung cancer. Cancer Res.

[56] Sun C (2015). Disturbance of redox status enhances radiosensitivity of hepatocellular carcinoma. Am J Cancer Res.

[57] Cong ZX (2014). Temozolomide and irradiation combined treatment-induced Nrf2 activation increases chemoradiation sensitivity in human glioblastoma cells. J Neurooncol.

[58] Jeong Y (2017). Role of KEAP1/NRF2 and TP53 Mutations in Lung Squamous Cell Carcinoma Development and Radiation Resistance. Cancer Discov.

[59] Ben-Cherif W (2013). Circadian variation of Valproic acid pharmacokinetics in mice. Eur J Pharm Sci.

[60] Powell-Jackson PR, Tredger JM, Williams R (1984). Hepatotoxicity to sodium valproate: a review. Gut.

[61] Saha, S. K. *et al*. Valproic Acid Induces Endocytosis-Mediated Doxorubicin Internalization and Shows Synergistic Cytotoxic Effects in Hepatocellular Carcinoma Cells. *Int J Mol Sci* **18**, 10.3390/ijms18051048 (2017). 10.3390/ijms18051048 PMC5454960 28498322

[62] Yonai S (2008). Evaluation of beam wobbling methods for heavy-ion radiotherapy. Med Phys.

[63] Tomayko MM, Reynolds CP (1989). Determination of subcutaneous tumor size in athymic (nude) mice. Cancer Chemother Pharmacol.

[64] Shin SW (2017). Mechanism of the Antitumor and Radiosensitizing Effects of a Manganese Porphyrin, MnHex-2-PyP. Antioxid Redox Signal.

