# Publisher Correction: Valproic Acid Sensitizes Hepatocellular Carcinoma Cells to Proton Therapy by Suppressing NRF2 Activation

**DOI:** 10.1038/s41598-018-25326-7

**Published:** 2018-05-10

**Authors:** Jeong Il Yu, Changhoon Choi, Sung-Won Shin, Arang Son, Ga-Haeng Lee, Shin-Yeong Kim, Hee Chul Park

**Affiliations:** 1 Departments of Radiation Oncology, Samsung Medical Center, Seoul, 06351 South Korea; 2 Sungkyunkwan University School of Medicine, Seoul, 06351 South Korea; 3 Department of Medical Device Management and Research, SAIHST, Seoul, 06351 South Korea

Correction to: *Scientific Reports* 10.1038/s41598-017-15165-3, published online 08 November 2017

In the original version of this Article, the author Jeong Il Yu was incorrectly indexed. This error has now been corrected.

