# Supplementary material for: Valproic Acid Sensitizes Hepatocellular Carcinoma Cells to Proton Therapy by Suppressing NRF2 Activation
Source: Sci Rep. 2017 Nov 8;7:14986. doi: 10.1038/s41598-017-15165-3 (PMC5678087; doi:10.1038/s41598-017-15165-3)
Supplement: Supplementary file 1 — Supplementary information [file 41598_2017_15165_MOESM1_ESM.pdf]

Supplementary information

Valproic Acid Sensitizes Hepatocellular Carcinoma Cells to Proton Therapy by Suppressing NRF2 Activation

Jeong Il Yu[1,*], Changhoon Choi[1,*], Sung-Won Shin[1,2], Arang Son[1], Ga-Haeng Lee[1], Shin-Yeong Kim[1] and Hee Chul Park[1,2.3]

[1]Departments of Radiation Oncology, Samsung Medical Center, [2]Sungkyunkwan University School of Medicine, [3]Department of Medical Device Management and Research, SAIHST, Seoul, 06351, South Korea.

*These authors contributed equally to this work.

Correspondence and requests for materials should be addressed to H.C.P. (email: hee.ro.park@samsung.com)

Figure 1a

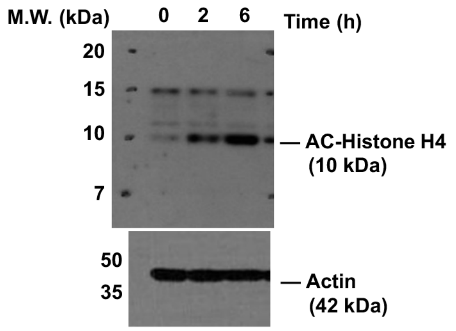

Figure 1b

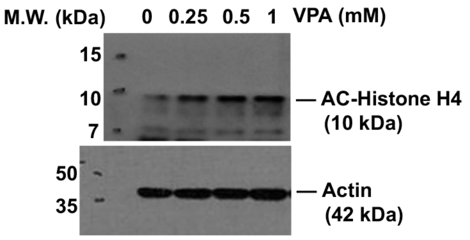

Figure 4c

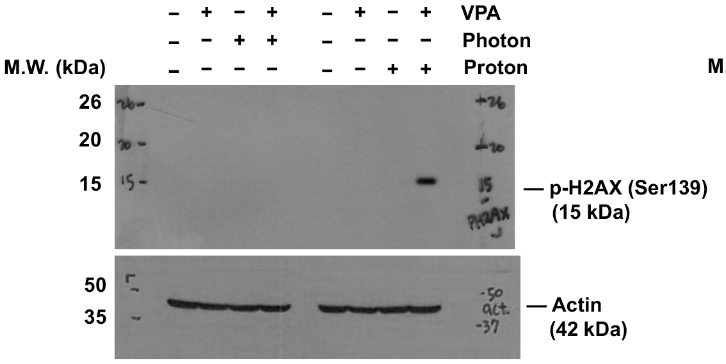

Figure 4d

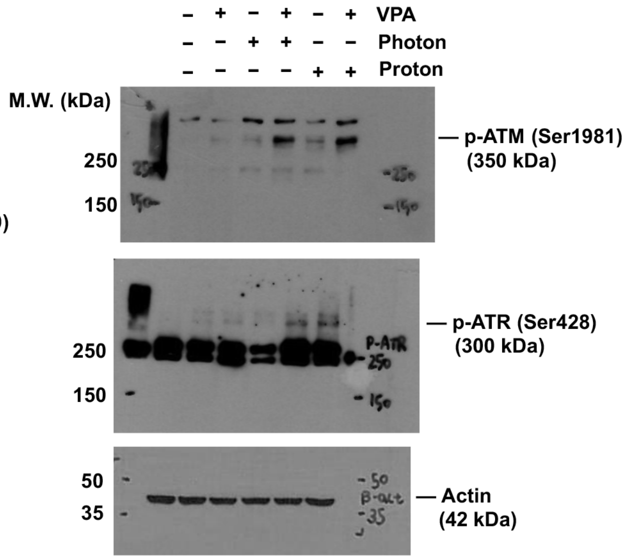

Figure 5b

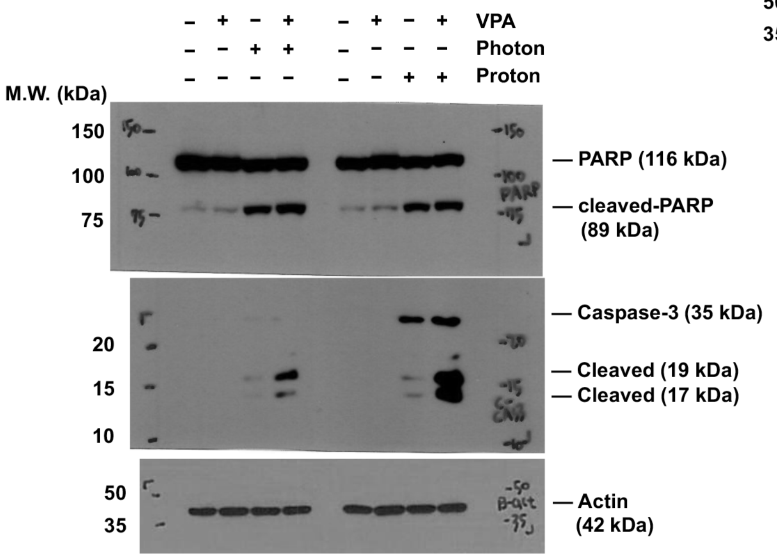

Figure 6b

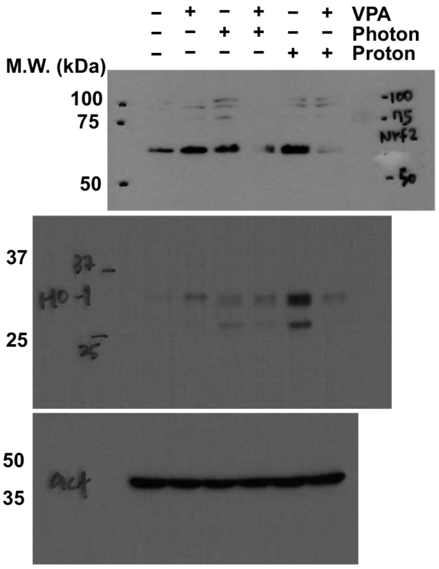

Figure 6c

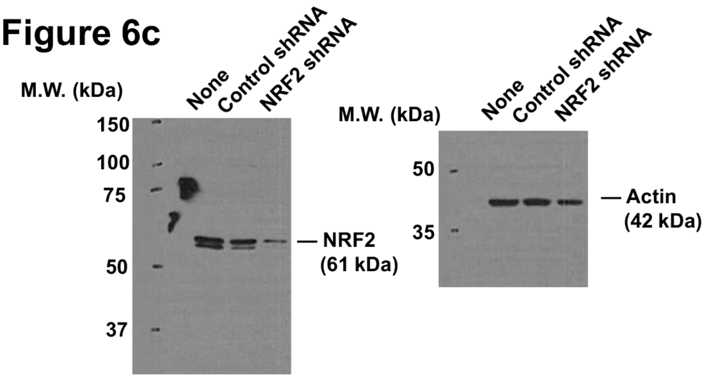

Supplementary figure S1. Full-length scans of the western blots presented in Figure 1a, 1b, 4c, 4d, 5b, 6b and 6c.
